# Supplementary material for: Evaluation of Cas13d as a tool for genetic interaction mapping
Source: Nat Commun. 2025 Feb 14;16:1631. doi: 10.1038/s41467-025-56747-4 (PMC11828948; doi:10.1038/s41467-025-56747-4)
Supplement: Supplementary file 1 — Supplementary Information [file 41467_2025_56747_MOESM1_ESM.pdf]

Cas13d as a tool for genetic interaction mapping

Ghanem El Kassem [1], Jasmine Hillmer [1], Michael Boettcher [1#]

[1] Universitätsmedizin Halle, Martin Luther University Halle-Wittenberg, Halle (Saale), 06120, Halle, Germany

# Corresponding author: Michael Boettcher
Email: michael.boettcher@medizin.uni-halle.de

Supplementary figures

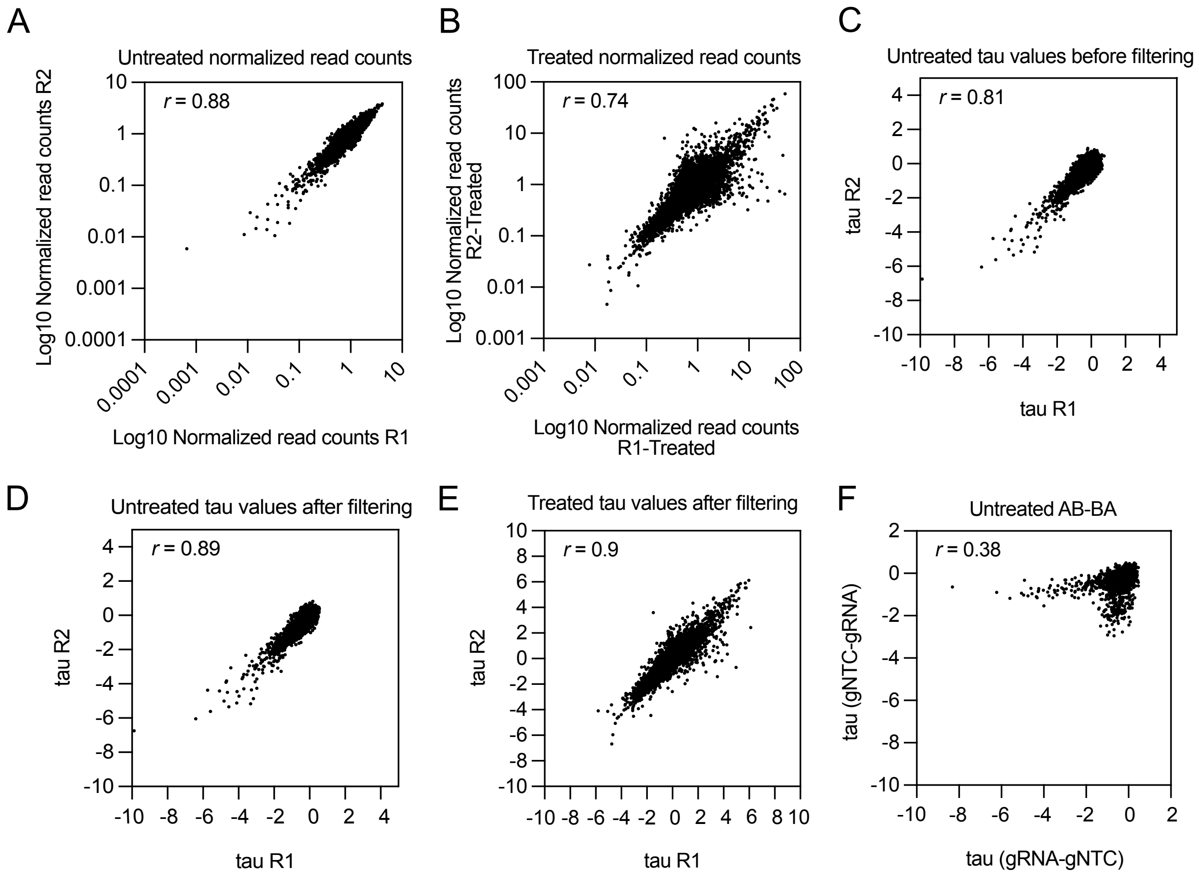

Supplementary Fig. 1 - High technical reproducibility of the U6-g1-g2 screens in K562 at different levels of data analysis. (A-B) Correlation between normalized read counts from two technical screen replicates in the untreated **(A)** and imatinib treated **(B)** conditions. **(C)** Correlation between tau values from two technical screen replicates in the untreated condition before filtering for functional gRNAs. **(D-E)** Correlation between tau values from two technical screen replicates in the untreated **(D)** and imatinib treated **(E)** condition after filtering for functional gRNAs. **(F)** Correlation between tau values from gRNA-gNTC and gNTC-gRNA combinations in the untreated condition. Pearson correlation was used to determine the *r* values. Source data are provided as a Source Data file.

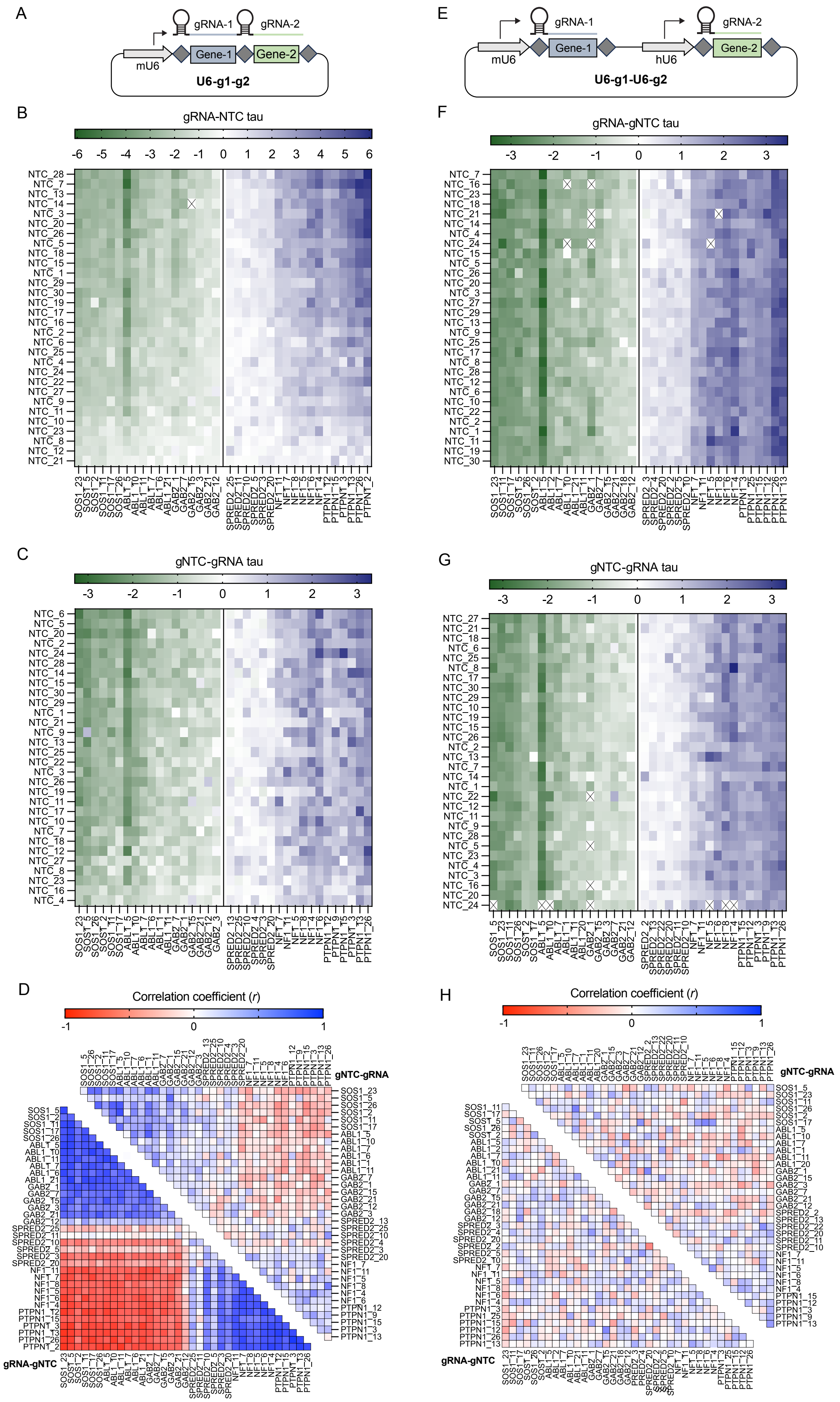
A
gRNA-1
gRNA-2
mU6
Gene-1
Gene-2
U6-g1-g2
B
gRNA-NTC tau
-6 -5 -4 -3 -2 -1 0 1 2 3 4 5 6
C
gNTC-gRNA tau
-3 -2 -1 0 1 2 3
D
Correlation coefficient (r)
-1 0 1
gNTC-gRNA
gRNA-gNTC
E
gRNA-1
gRNA-2
mU6
hU6
Gene-1
Gene-2
U6-g1-U6-g2
F
gRNA-gNTC tau
-3 -2 -1 0 1 2 3
G
gNTC-gRNA tau
-3 -2 -1 0 1 2 3
H
Correlation coefficient (r)
-1 0 1
gNTC-gRNA
gRNA-gNTC

Supplementary Fig. 2 - Concatenated Cas13d gRNAs show sequence-dependent interference which is overcome by gRNA expression from separate promoters. (A) Schematic of the dual gRNA concatenation strategy U6-g1-g2. mU6 = mouse U6 promoter, diamonds = direct repeat DR36, squares = target-specific spacer sequence. **(B)** Heatmap showing tau values from the 6 best performing gene-targeting gRNAs in position g1 (x-axis) combined in one array with 30 different non-target control gRNA sequences (gNTC) in position g2 (y-axis) using the U6-g1-g2 strategy. **(C)** Heatmap showing tau values from the 6 best performing gene-targeting gRNAs (x-axis) in position g2, combined in one array with 30 different gNTCs in position g1 (y-axis) using the U6-g1-g2. **(D)** Pearson correlation (*r*) between tau values from all columns in (B) (lower left) and (C) (upper right). **(E)** Schematic of the dual promoter gRNA expression strategy U6-g1-U6-g2. mU6 = mouse U6 promoter, hU6 = human U6 promoter, diamonds = direct repeat DR36, squares = target-specific spacer sequence. **(F)** Heatmap of tau values from gene-targeting gRNAs expressed from the mU6 promoter co-expressed with indicated gNTCs from hU6. **(G)** Heatmap of tau values from gene-targeting gRNAs expressed from the hU6 promoter co-expressed with indicated gNTCs from mU6. **(H)** Pearson correlation (*r*) between tau values from all columns in (F) (lower left) and (G) (upper right). Source data are provided as a Source Data file.

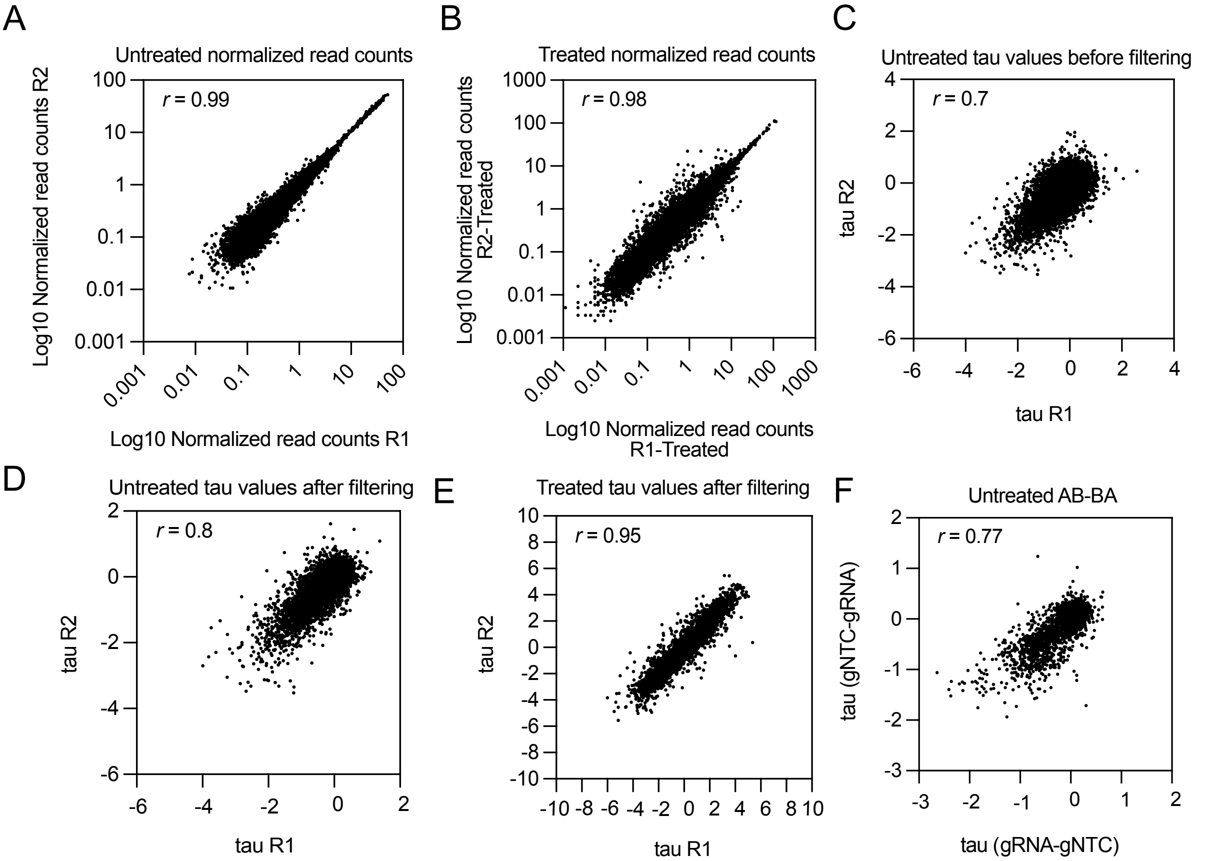

Supplementary Fig. 3 - High technical reproducibility of the U6-g1-U6-g2 screens in K562 at different levels of data analysis. (A-B) Correlation between normalized read counts from two technical screen replicates in the untreated **(A)** and imatinib treated **(B)** conditions. **(C)** Correlation between tau values from two technical screen replicates in the untreated condition before filtering for functional gRNAs. **(D-E)** Correlation between tau values from two technical screen replicates in the untreated **(D)** and imatinib treated **(E)** condition after filtering for functional gRNAs. **(F)** Correlation between tau values from gRNA-gNTC and gNTC-gRNA combinations in the untreated condition. Pearson correlation was used to determine the *r* values. Source data are provided as a Source Data file.

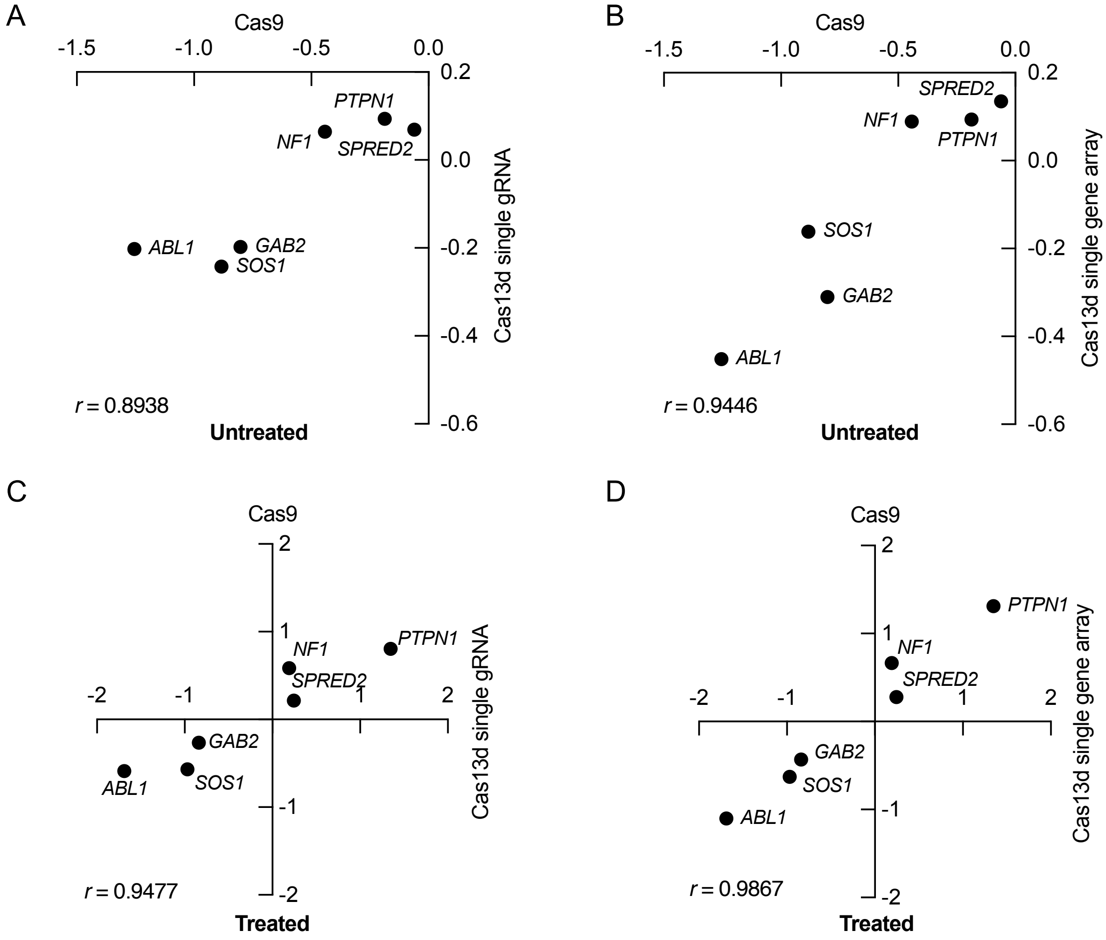

E

Gene	DepMap Chronos	Untreated beta score: Cas9	Untreated beta score: Single gRNA	Untreated beta score: Single gene array	Treated beta score: Cas9	Treated beta score: single gRNA	Treated beta score: single gene array
ABL1	-1.63	-1.25	-0.20	-0.45	-1.69	-0.59	-1.10
GAB2	-1.69	-0.80	-0.20	-0.31	-0.84	-0.27	-0.43
NF1	-0.30	-0.44	0.06	0.09	0.19	0.59	0.67
PTPN1	-0.91	-0.19	0.09	0.09	1.35	0.80	1.31
SOS1	-1.37	-0.88	-0.24	-0.16	-0.97	-0.57	-0.63
SPRED2	0.04	-0.06	0.07	0.14	0.24	0.22	0.28

F

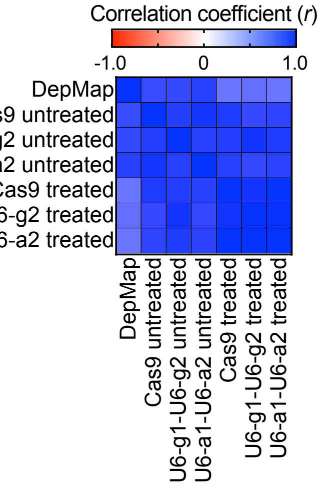

Supplementary Fig. 4 - Cas13d shows no signs of unspecific growth phenotypes. (A) Correlation between MAGeCK MLE beta scores of the untreated Cas9 counter screen and single gRNA screen (U6-g1-U6-g2). **(B)** Correlation between MAGeCK MLE beta scores of the untreated Cas9 counter screen and the single-gene array screen (U6-a1-U6-a2). **(C)** Correlation between MAGeCK MLE beta scores of the imatinib treated Cas9 counter screen and single gRNA screen (U6-g1-U6-g2). **(D)** Correlation between MAGeCK MLE beta scores of the imatinib treated Cas9 counter screen and single-gene array screen (U6-a1-U6-a2). **(E)** Table of the DepMap Chronos scores of the 6 candidate genes in K562 and the MAGeCK MLE beta scores of the Cas9 counter screens and all Cas13d screens. **(F)** Pearson correlation (*r*) between beta scores shown in (E). Source data are provided as a Source Data file.

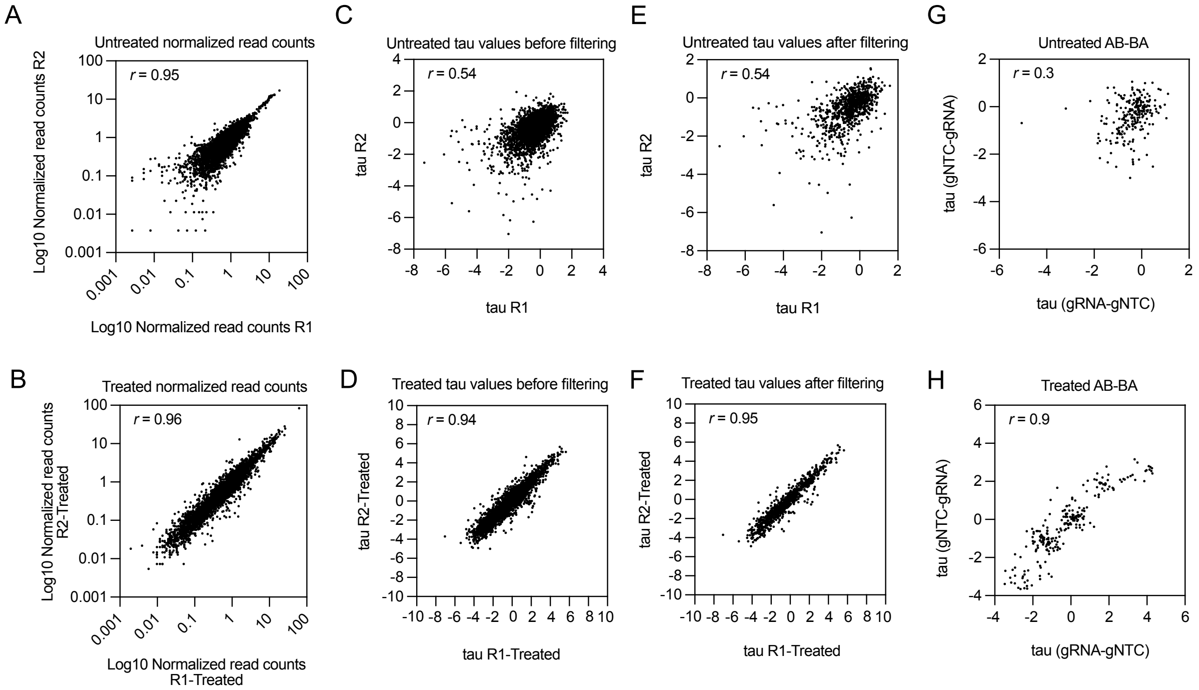

Supplementary Fig. 5 - High technical reproducibility of the U6-a1-U6-a2 screens in K562 at different levels of data analysis. (A-B) Correlation between normalized read counts from two technical screen replicates in the untreated **(A)** and imatinib treated **(B)** conditions. **(C-D)** Correlation between tau values from two technical screen replicates in the untreated **(C)** and imatinib treated **(D)** condition before filtering for functional gRNAs. **(E-F)** Correlation between tau values from two technical screen replicates in the untreated **(D)** and imatinib treated **(E)** condition after filtering for functional gRNAs. **(G-H)** Correlation between tau values from gRNA-gNTC and gNTC-gRNA combinations in the untreated **(G)** and imatinib treated **(H)** condition. Pearson correlation was used to determine the *r* values. Source data are provided as a Source Data file.

A

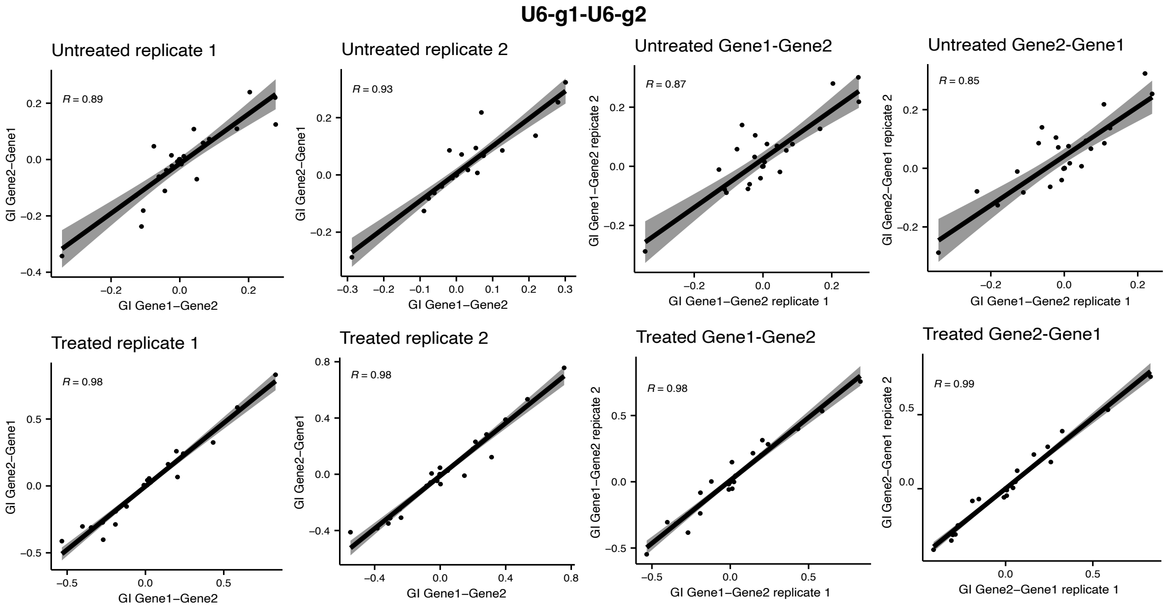

B

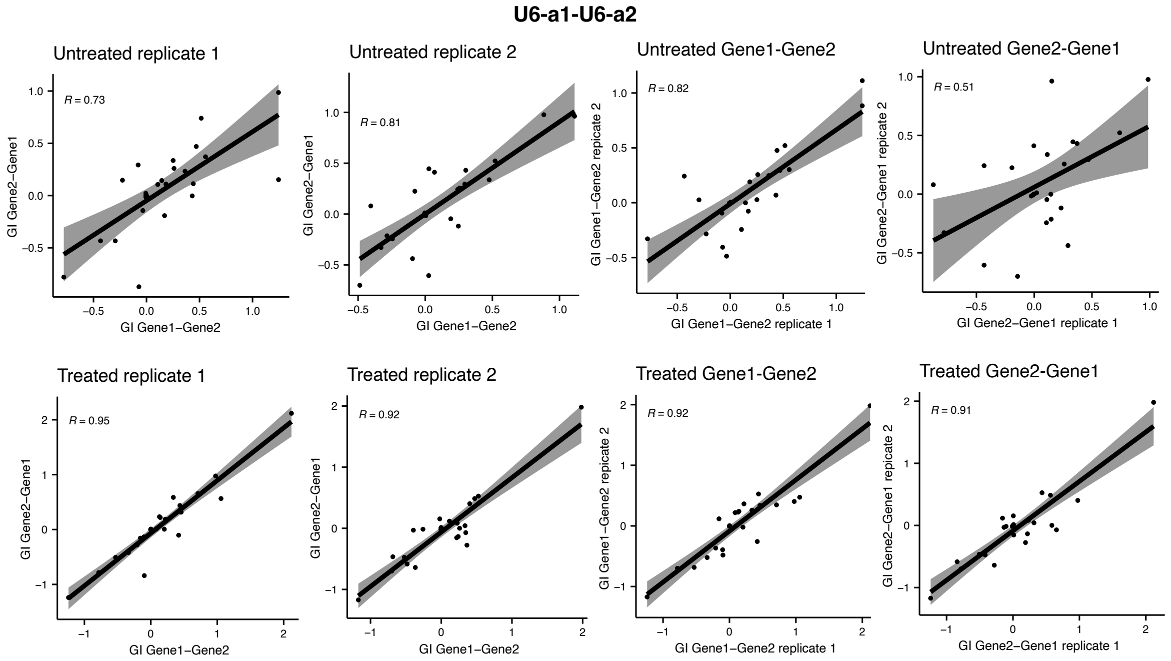

Supplementary Fig. 6 - Cas13d enables the highly reproducible identification of GIs. Pearson correlation between GI scores determined from **(A)** single gRNA and **(B)** single-gene array screens, without (top) and with imatinib treatment (bottom). The left panels show the correlation of GI scores between Gene1-Gene2 and Gene2-Gene1 orientations. The right panels show the correlation of GI scores between screen replicates. Source data are provided as a Source Data file.

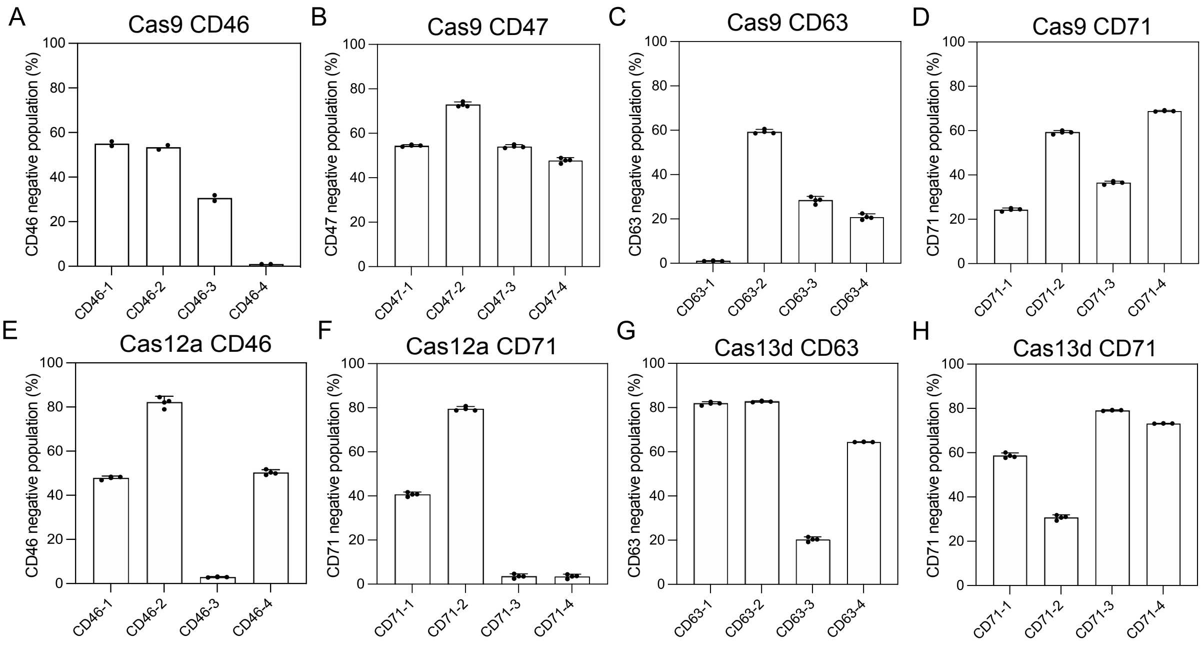

Supplementary Fig. 7 - Knockout efficiency of Cas9 and Cas12a sgRNAs and Cas13d arrays per gene targeting different cellular surface markers. (A-H) Percentage of gene knockout cells using the indicated CRISPR system was determined via flow cytometry analysis of >10,000 cells stained with CD46-APC antibodies (Miltenyi). **(A)** CD46 knockout with 4 Cas9 sgRNAs in HEK293ΔRAF1:ER cells (47). **(B)** CD47 knockout with 4 Cas9 sgRNAs in K562 cells. **(C)** CD63 knockout with 4 Cas9 sgRNAs in K562 cells. **(D)** CD71 knockout with 4 Cas9 sgRNAs in K562 cells. **(E)** CD46 knockout with 4 Cas12a gRNAs in K562 cells. **(F)** CD71 knockout with 4 Cas12a gRNAs in K562 cells. **(G)** CD63 knockout with 4 Cas13d arrays in K562 cells. **(H)** CD71 knockout with 4 Cas13d arrays in K562 cells. Values represent the mean of biological replicates; error bars, SD (n=3 except in (A) where n=2). Source data are provided as a Source Data file.
