# Supplementary material for: Evaluation of Cas13d as a tool for genetic interaction mapping
Source: Nat Commun. 2025 Feb 14;16:1631. doi: 10.1038/s41467-025-56747-4 (PMC11828948; doi:10.1038/s41467-025-56747-4)
Supplement: Supplementary file 2 — Description of Additional Supplementary Files [file 41467_2025_56747_MOESM2_ESM.pdf]

Description of Additional Supplementary Files

File Name: Supplementary Data 1
Description: List of gRNA and array sequences in Cas9 and Cas13d libraries.

File Name: Supplementary Data 2
Description: Calculated tau values and GI scores of gRNA and array sequences from samples of Cas9 and Cas13d screens.

File Name: Supplementary Data 3
Description: List of gRNA sequences, primers, and DNA fragments.

File Name: Supplementary Data 4
Description: Statistical analysis of GI scores calculated from Cas13d screens.

File Name: Supplementary Data 5
Description: Raw count numbers of gRNA and array sequences from samples of Cas9 and Cas13d screens.
